# CLEAR – clozapine in early psychosis: study protocol for a multi-centre, randomised controlled trial of clozapine vs other antipsychotics for young people with treatment resistant schizophrenia in real world settings

**DOI:** 10.1186/s12888-023-05397-1

**Published:** 2024-02-14

**Authors:** C. Casetta, P. Santosh, R. Bayley, J. Bisson, S. Byford, C. Dixon, R. J. Drake, R. Elvins, R. Emsley, N. Fung, D. Hayes, O. Howes, A. James, K. James, R. Jones, H. Killaspy, B. Lennox, L. Marchant, P. McGuire, E. Oloyede, M. Rogdaki, R. Upthegrove, J. Walters, A. Egerton, J. H. MacCabe

**Affiliations:** 1 Institute of Psychiatry, Psychology & Neuroscience, King’s College London, London, UK; 2 South London and Maudsley NHS Foundation Trust, London, UK; 3 Division of Psychological Medicine and Clinical Neurosciences, Cardiff University, Cardiff, UK; 4 Wonford House Hospital, Devon Partnership NHS Trust, Exeter, UK; 5 Division of Psychology & Mental Health, University of Manchester, Manchester, UK; 6 Greater Manchester Mental Health NHS Foundation Trust, Manchester, UK; 7 Manchester University Hospitals NHS Foundation Trust, Manchester, UK; 8 Birmingham Women’s and Children’s NHS Foundation Trust, Birmingham, UK; 9 Department of Psychiatry, University of Oxford, Oxford, UK; 10 Birmingham and Solihull Mental Health Foundation Trust, Birmingham, UK; 11 Division of Psychiatry, University College London, London, UK; 12 Institute for Mental Health, University of Birmingham, Birmingham, UK; 13 Birmingham Early Intervention Service, Birmingham Womens and Childrens NHS Foundation Trust, Birmingham, UK

**Keywords:** Clozapine, Treatment resistant psychosis, Children and young people, Early onset schizophrenia, Clinical trial

## Abstract

**Background:**

Clozapine is an antipsychotic drug with unique efficacy, and it is the only recommended treatment for treatment-resistant schizophrenia (TRS: failure to respond to at least two different antipsychotics). However, clozapine is also associated with a range of adverse effects which restrict its use, including blood dyscrasias, for which haematological monitoring is required. As treatment resistance is recognised earlier in the illness, the question of whether clozapine should be prescribed in children and young people is increasingly important. However, most research to date has been in older, chronic patients, and evidence regarding the efficacy and safety of clozapine in people under age 25 is lacking. The CLEAR (CLozapine in EARly psychosis) trial will assess whether clozapine is more effective than treatment as usual (TAU), at the level of clinical symptoms, patient rated outcomes, quality of life and cost-effectiveness in people below 25 years of age. Additionally, a nested biomarker study will investigate the mechanisms of action of clozapine compared to TAU.

**Methods and design:**

This is the protocol of a multi-centre, open label, blind-rated, randomised controlled effectiveness trial of clozapine vs TAU (any other oral antipsychotic monotherapy licenced in the British National Formulary) for 12 weeks in 260 children and young people with TRS (12–24 years old).

**Aim and objectives:**

The primary outcome is the change in blind-rated Positive and Negative Syndrome Scale scores at 12 weeks from baseline. Secondary outcomes include blind-rated Clinical Global Impression, patient-rated outcomes, quality of life, adverse effects, and treatment adherence. Patients will be followed up for 12 months and will be invited to give consent for longer term follow-up using clinical records and potential re-contact for further research. For mechanism of action, change in brain magnetic resonance imaging (MRI) biomarkers and peripheral inflammatory markers will be measured over 12 weeks.

**Discussion:**

The CLEAR trial will contribute knowledge on clozapine effectiveness, safety and cost-effectiveness compared to standard antipsychotics in young people with TRS, and the results may guide future clinical treatment recommendation for early psychosis.

**Trial registration:**

ISRCTN Number: 37176025, IRAS Number: 1004947.

**Trial status:**

In set-up. Protocol version 4.0 01/08/23. Current up to date protocol available here: https://fundingawards.nihr.ac.uk/award/NIHR131175#/.

## Introduction

Treatment resistant schizophrenia (TRS), defined by NICE and most other treatment guidelines as non-response to at least two different antipsychotic drugs (at least 1 of which should be a non‑clozapine second‑generation antipsychotic) [1], affects around one third of people diagnosed with schizophrenia [2]. TRS is associated with severe long-term consequences on social, educational and occupational functioning, with total treatment costs between three and eleven times that of schizophrenia that is responsive to standard treatment [3]. Treatment resistance in schizophrenia is strongly associated with age at illness onset, with onset before age 25 predicting higher risk of subsequent treatment resistance [4]. Clozapine is the only antipsychotic to have superior efficacy in TRS and is the treatment of choice in adults with TRS [5]. This is supported by evidence from randomised controlled trials [6, 7] although some doubt has been cast on the strength of this evidence [8]. Pharmacoepidemiological studies have demonstrated the superiority of clozapine over other antipsychotics in reducing readmissions [9], violent offending [10] self-harm [11] and all-cause mortality [12].

Most of the evidence for the superiority of clozapine over other antipsychotics derives from studies in chronic patients; a recent meta-analysis found the median age in trials of clozapine to be 39 years with a median length of illness of 16 years [8]. Delay in initiating clozapine is associated with poorer response, and earlier use of clozapine and fewer pre-clozapine antipsychotic trials have been found to be associated with better treatment outcomes [13, 14]. The evidence for the efficacy of clozapine in younger patients is sparse, although it suggests that clozapine is superior to other antipsychotics in people under 18 with TRS [15], and that younger age is associated with greater symptom reduction in clozapine-treated people [16]. A recent retrospective study showed that the vast majority of paediatric patients (95%) admitted with or started on clozapine during an acute psychiatric hospitalization remained on clozapine at discharge, suggesting that it may be clinically effective [17]. Furthermore, a Danish cohort study on early onset schizophrenia showed that the majority of patients (88.8%) prescribed clozapine were considered as having a favourable response since they continued to redeem clozapine prescriptions for more than 6 months [18]. However, there are significant limitations of naturalistic, observation data such as these, including that clinicians may be unlikely to stop clozapine treatment once started given a perception it is a treatment of last resort [19]. There is thus a clear need for larger scale clinical trials in younger patients.

Clozapine is reserved as a third line treatment because of its associated adverse effects, which are more numerous and severe than those of most other antipsychotics [20]. The most problematic is the rare but potentially fatal adverse effect of severe neutropenia [21]. In order to reduce the risk of agranulocytosis, in the UK and most other Western countries, monitoring of the patient’s full blood count is mandatory in clozapine-treated patients [22]. There is evidence that psychiatrists unfamiliar with clozapine are reluctant to prescribe it, and that blood testing in particular acts as a barrier [23]. This may particularly apply to child and adolescent psychiatrists, who rarely encounter TRS. Less than 0.5% of prescriptions for clozapine are in children and adolescents, and a survey of UK psychiatrists showed that only 40% of psychiatrists working in UK Child and Adolescent services have ever prescribed clozapine [24]. The probable superior efficacy of clozapine in younger patients must be balanced against its potentially inferior tolerability [25]. A recent literature review concludes that the risk–benefit ratio for clozapine use in young TRS patients is unclear, and that the question can only be resolved by conducting well powered studies that simultaneously measure safety and effectiveness [26].

The NICE guidance for schizophrenia and psychosis in adults (CG-178) and children (CG-155) recommend clozapine in patients whose illness has not responded to trials of at least two antipsychotics of adequate dose and duration. Nevertheless, the NICE Guideline Development Group (NICE Recommendation CG155/5) and the James Lind Alliance [27] have both identified the lack of evidence surrounding this recommendation, particularly with regard to overall cost-effectiveness. A well powered randomised controlled effectiveness trial is required to fill this gap. No such study has been completed to date, probably reflecting the difficulties of recruitment in this patient group. This trial will assess whether clozapine is more effective than treatment as usual (TAU: standard antipsychotics) in people below 25 years of age, at the level of clinical symptoms, patient rated outcomes, quality of life and cost-effectiveness.

The mechanisms underlying the efficacy of clozapine in TRS are not well understood [28]. Dopamine D2 receptor antagonism is the key mediator of efficacy for non-clozapine antipsychotics, but clozapine is a relatively weak dopamine D2 receptor antagonist, suggesting that its mode of action lies elsewhere [29]. Leading theories of schizophrenia pathogenesis include the interlinked processes of increased inflammation, oxidative stress and glutamate release [30, 31]. Preclinical research has shown that, compared to most other antipsychotics, clozapine may be particularly effective in reducing expression of proinflammatory cytokines (e.g. IL-6), increasing expression of anti-inflammatory cytokines (e.g. IL-10) [32, 33], has greater antioxidant effects [34–36], and can reduce brain glutamate [37–39]. Higher brain glutamate metabolite levels have been found in studies of patients with TRS [40–42], and reductions in glutamate have been observed during clozapine treatment [43]. The trial will incorporate a mechanistic study to investigate whether the efficacy of clozapine compared to TAU is linked to its actions on these pathways.

## Trial design

This is a multi-centre, open label, blind-rated, randomised, controlled effectiveness trial of clozapine vs TAU (i.e., compared with other antipsychotics of clinician’s choice) for 12 weeks in 260 children and young people with TRS (12–24 years old).

### Aim and objectives

The purpose of the trial is to assess whether clozapine is more effective than TAU (standard antipsychotics) in young people with TRS in real-world settings, over a 12-week period, at the level of clinical symptoms, patient-rated outcomes, quality of life and cost-effectiveness. The primary objective is to compare the treatments on the change in total Positive and Negative Syndrome Scale (PANSS) [44] score from baseline to 12 weeks. The secondary objectives are to compare the treatments on function, adverse effects, quality of life, subjective improvement, and cost-effectiveness.

In the CLEAR trial the following hypotheses will be tested:

- Primary hypothesis: clozapine will be more effective than TAU after 12 weeks of treatment as demonstrated by reduction in PANSS total scores
- Secondary hypotheses:
  1. Clozapine will lead to a higher global improvement than TAU as measured by the CGI at 12 weeks, 24 weeks, and 52 weeks
  2. Clozapine will lead to a better improvement in health-related quality of life than TAU as measured by EQ-5D-Y at 12 weeks, 24 weeks, and 52 weeks
  3. Clozapine will be cost-effective compared to TAU at 12 weeks, 24 weeks, and 52 weeks
  4. The prevalence of adverse effects in participants on the clozapine arm will be higher than TAU at 12 weeks as measured by the GASS-C, weight, and metabolic laboratory measurements (i.e., higher HbA1C and lipids)
  5. The clozapine arm will be more likely to remain on the same medication longer-term than TAU, as measured by medication use at 24 weeks and 52 weeks.
- Mechanistic hypotheses:
  - Improvement in schizophrenia symptom severity during clozapine treatment compared to TAU will be mediated by anti-inflammatory and glutamatergic mechanisms. The biomarkers characterising these anti-inflammatory and glutamatergic mechanisms will include reductions in peripheral proinflammatory cytokines (e.g., IL-6), brain glutamate and rCBF, and increases in anti-inflammatory cytokines (e.g., IL-10) and peripheral and central oxidative defence (GSH) from baseline to 12 weeks.

### Trial flowchart

**Fig. 1:**
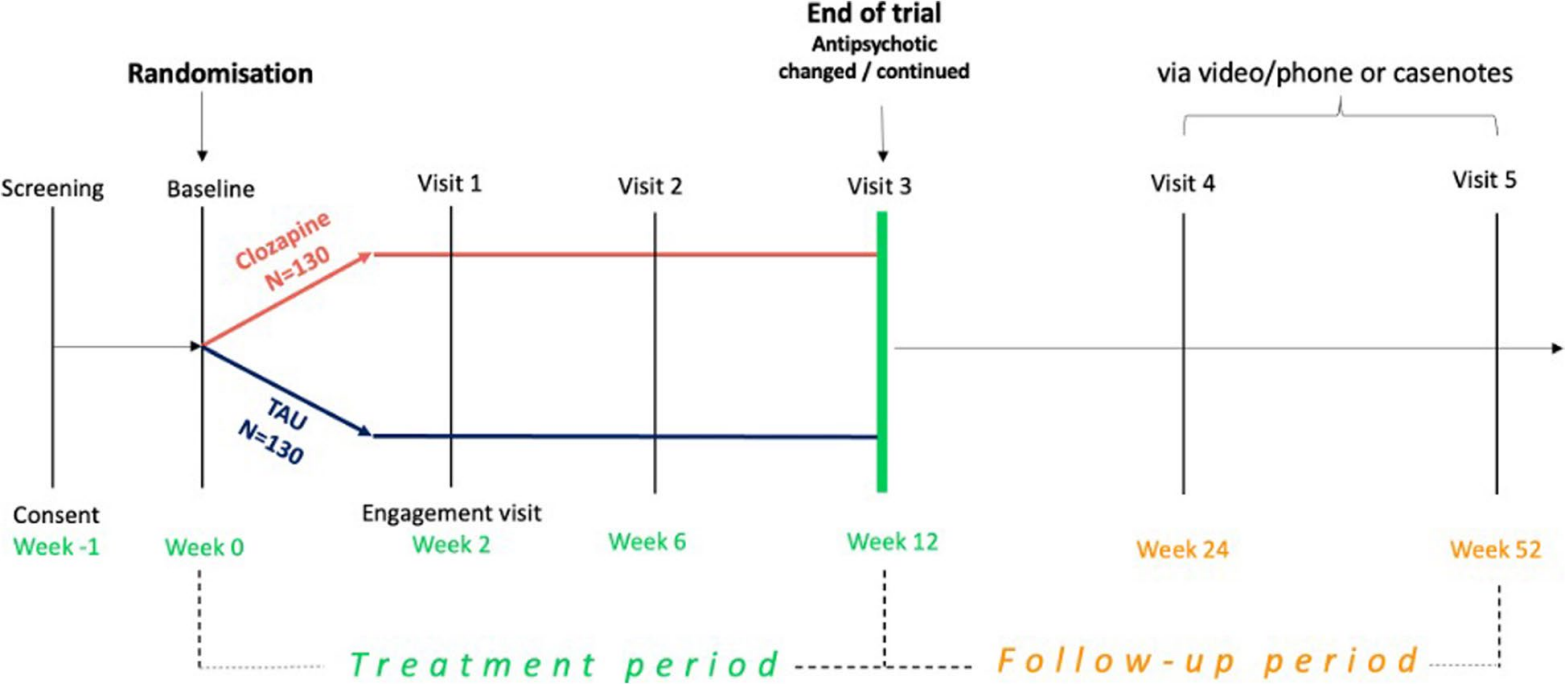
Trial flowchart

**Fig. 2:**
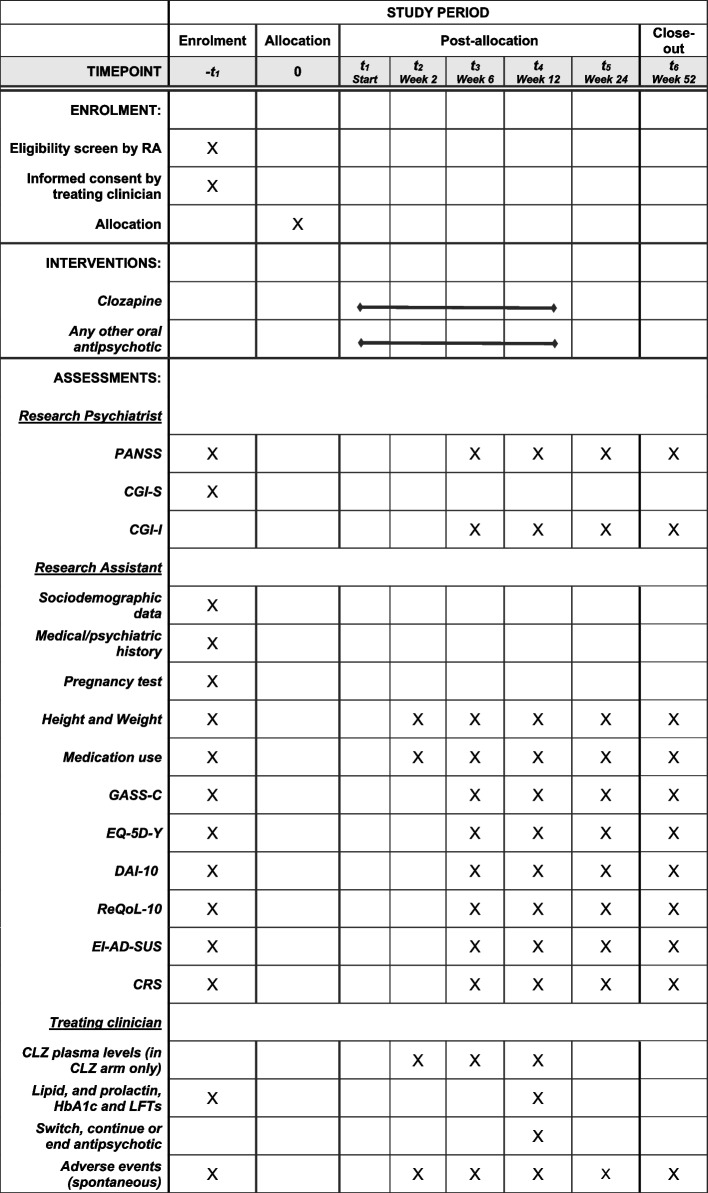
SPIRIT flow diagram: Schedule of enrolment, interventions, and assessments. *RA*: Research Assistant; *CLZ*: clozapine; *PANSS*: Positive and Negative Syndrome Scale; *CGI-S*: Clinical Global Impression – Severity; *CGI-I*: Clinical Global Impression – Improvement; *GASS-C*: Glasgow Antipsychotic Side-effects Scale for Clozapine; *EQ-5D-Y*: Youth version of the EQ-5D; *DAI-10*: Drug Attitude Inventory–10 items; *ReQoL-10*: Recovering Quality of Life Questionnaire; *EI-AD-SUS*: Early Intervention Adult Service Use Schedule; *CRS*: Clinician Rating Scale

Figure 1 shows the trial flowchart. During the clinical trial (treatment period), participants will be allocated to either clozapine or TAU. After 12 weeks of treatment, participants will be able to continue or change treatment according to clinical needs and existing guidelines, and they will be followed up via phone or videocall whenever possible, otherwise case notes will be reviewed to assess longer-term outcomes. Schedule of enrolment, interventions and assessments are shown in the SPIRIT flow diagram (Fig. 2).

### Setting

We will recruit from NHS-funded secondary care, both inpatient and community settings, using a hub and spoke model, with 6 academic centres (King’s College London, University of Birmingham, Cardiff University, The University of Manchester, University of Oxford, University College London) coordinating approximately 30 NHS recruitment sites.

### Study population and eligibility criteria

We will recruit 260 children and young people from 12 to 24 years old with TRS as defined by NICE (CG178, Sect. 1.5.7.2). Inclusion and exclusion criteria are specified in greater detail in Table 1.

**Table 1:** Inclusion and exclusion criteria

**Inclusion criteria**	**Exclusion criteria**
1. Age ≥ 12 and < 25 years at randomization 2. Meets criteria for schizophrenia or related disorder, in the range in the range ICD-10v2016 F20.x, F22.x-F29.x 3. Meets criteria for treatment resistance, defined as: a. Previous trials of at least two different antipsychotic drugs with adequate adherence (estimated < 20% missed doses), both treatment trials to exceed 6 weeks at therapeutic dose (≥ 600 mg chlorpromazine equivalent) b. At least 1 of these trials must be with a second-generation drug c. Failure to respond to NICE-recommended psychological treatment OR failure to engage in same 4. PANSS total ≥ 70, at least 2 items > 4 5. CRS > 3 6. Capacity to give informed consent OR has a legal representative able to give consent to the trial 7. English or Welsh language sufficient to participate	1. Psychosis predominantly caused by substance misuse 2. Pregnancy 3. Breastfeeding 4. Contra-indications to clozapine as listed in SmPC as follows: a. Hypersensitivity to the active substance or to any of the excipients b. Patients unable to undergo regular blood tests c. History of toxic or idiosyncratic granulocytopenia/ agranulocytosis (except for granulocytopenia/ agranulocytosis from previous chemotherapy) d. History of clozapine-induced agranulocytosis e. Impaired bone marrow function f. Uncontrolled epilepsy g. Alcoholic and other toxic psychoses, drug intoxication, comatose conditions h. Circulatory collapse and/or CNS depression of any cause i. Severe renal or cardiac disorders (e.g., myocarditis) j. Active liver disease associated with nausea, anorexia or jaundice, progressive liver disease, hepatic failure k. Paralytic ileus l. Clozapine treatment must not be started concurrently with substances known to have a substantial potential for causing agranulocytosis; concomitant use of depot antipsychotics is to be discouraged 5. Previous adequate trial of clozapine, as defined by TRIPP working group consensus guidelines, i.e. duration of at least 3 months following attainment of therapeutic plasma levels or a minimum dose of 500 mg/day (if no plasma level available) 6. CNS disorders (ICD-10 G00-26; G40-41, G45-46; G80-94, G97) 7. Concurrent medications with documented interactions with antipsychotics^a^ 8. Participation in a medicinal trial involving an unlicensed, investigational medical product within the last 3 month 9. Positive test for COVID-19 within the past 10 days 10. For participation in the substudy MRI scan only, standard contraindications to MRI at 3 Tesla such as ferromagnetic or electronic implants

*PANSS *Positive and Negative Syndrome Scale,* CRS *Clinician Rating Scale

^a^Defined as (1) strong Cytochrome P-450 CYP1A2 Inhibitors (i.e. fluvoxamine, abiraterone, midostaurin, enoxacin, ciprofloxacin, zafirlukast, Technetium Tc-99 m ciprofloxacin, furafylline, rofecoxib, quinidine, clinafloxacin, amiodarone, viloxazine) [45]; or (2) life-threatening or contraindicated combination (i.e. abametapir, domperidone, hydroxyzine, mizolastine, nirmatrelvir, ritonavir, sibutramine) [46]

### Interventions

During the 12 weeks of the trial, we will compare clozapine vs. TAU.

Intervention arm: Clozapine, oral, flexible dose within dose range defined by British National Formulary [47], at the discretion of the prescriber, for a minimum of 12 weeks. As clozapine has unpredictable pharmacokinetics with high heterogeneity in plasma concentration, depending on age, sex, smoking status, and genetics of the liver enzymes that metabolise it (especially CYP-1A2 and-2D6), enforcing a fixed dose would reduce the acceptability of the trial to patients and clinicians. It also requires titration over the first 2 weeks up to therapeutic doses to minimise postural hypotension, and the optimal balance between efficacy and adverse effects can only be achieved on an individual basis. The previously prescribed antipsychotic can be titrated down during the first 2 weeks of clozapine treatment and must be stopped within the first two weeks.

Control arm: Any oral antipsychotic other than clozapine, within licensed dose range defined by BNF [47], for a minimum of 12 weeks. The choice of antipsychotic will be agreed by the clinical team in collaboration with the participant, and the dose titrated to achieve the best balance between response and adverse effects.

### Outcomes

The primary outcome is the change in total blind rated PANSS total scores at 12 weeks from baseline.

Secondary outcomes include: blind rated change in overall Clinical Global Impression Scale (CGI) [48], clinician rated level of adherence (CRS) [49], adverse effects (GASS-C) [50], health-related quality of life (EQ-5D-Y) [51], subjective experience (DAI-10) [52], readmission rate, (EI-AD-SUS) [53], and change in PANSS sub-scale (positive, negative and general) [44]. We will also combine these outcomes (EQ-5D-Y and total PANSS score) with service use data (EI-AD-SUS) to compare treatments on cost-effectiveness.

Patients will be followed up for 12 months with the potential for further follow-up using clinical records for longer-term evaluations.

For the mechanistic study, outcomes include the change in brain glutamate, regional cerebral blood flow (rCBF) and GSH, and peripheral cytokines and GSH from baseline to 12 weeks.

### Assessments

#### Clinical trial

Centralised, remote, blind assessments by Research Psychiatrists will include:

- PANSS, the primary outcome. It is the most well validated standardised rating scale in clinical trials of psychosis
- CGI. It is simple and designed to capture the overall clinical judgement of an experienced clinician. CGI-Severity (CGI-S) will be assessed at baseline, and CGI-Improvement (CGI-I) will be assessed in the following visits.

Data collected by Research Assistants (RAs) will include:

- Medical history: Full history of antipsychotic use, doses, and response
- Patient-rated outcome measure (PROM): Drug Attitude Inventory (DAI-10)
- Adverse events: Glasgow Antipsychotic Side-effect Scale for Clozapine (GASS-C). It is a modification of the GASS, a well validated side effect scale, with additional questions pertaining to common adverse effects of clozapine
- Adverse events: spontaneous report. The RAs will prompt for any other suspected adverse reactions and record these
- Adherence: Clinician Rating Scale (CRS)
- Health-related quality of Life: EQ-5D-Y, a questionnaire which is used to generate quality adjusted life years (QALYs) for use in economic evaluation, as recommended by NICE
- Service use for economic evaluation: Early Intervention Adult Service Use Schedule (EI-AD-SUS), a measure specifically designed for use in children and young adults with psychosis
- Recovering Quality of Life-10 items measure (ReQoL-10) [54], a measure of health-related quality of life specifically designed for users of mental health services.

Laboratory measurements will be collected at baseline and at 12 weeks. Blood tests include HbA1c, lipids, prolactin, and liver function tests. As per standard care, the clozapine arm will undergo weekly blood monitoring to check full blood count and clozapine plasma levels as per standard care.

#### Follow-up period

After the end of the trial, we will follow-up participants after 24 and 52 weeks via video-link or phone call; if this is not possible, we will retrieve information reviewing case notes. We will record medication use, spontaneous adverse events, PANSS score, CGI-I, GASS-C, EQ-5D-Y, CRS, DAI-10, ReQol-10, EI-AD-SUS.

#### Mechanistic study

Brain Magnetic Resonance Imaging (MRI) scans and blood samples will be acquired at baseline and 12 weeks. MRI data will be acquired at 3 Tesla at 3 centres, and will include proton magnetic resonance spectroscopy (1H-MRS) to measure levels of glutamate, GSH and other brain metabolites visible in the 1H-MRS spectrum in voxels positioned in the bilateral anterior cingulate cortex and right striatum, arterial spin labelling to measure rCBF and T1-weighted structural brain images to provide anatomical localisation, grey matter masking of rCBF images and correction of 1H-MRS data for voxel tissue composition. The blood samples will be used to measure levels of peripheral pro- and anti-inflammatory cytokines and GSH.

### Sample size

The standardised mean difference for clozapine versus other antipsychotics in adults based on PANSS total score at 12 weeks is 0.39 [55]. The standard deviation of the PANSS score at 12 weeks in our similar sample of treatment resistant patients being started on clozapine [43] is 14.2 (60% CI 12.96–16.20). Using an upper limit of the 60% confidence interval [56], an effect size of 0.39 corresponds to a between group difference of 6.4 points. In our previous research [43], the 95% CI for the baseline-12-week correlation in the sample was 0.553 to 0.880, and we use a value of 0.5. This is conservative but realistic, since the time points are only 12 weeks apart. Under these assumptions, and assuming 20% attrition to 12-week follow-up, the target sample size to recruit at baseline is 260 participants.

For the mechanistic study we estimate that, of all participants recruited at MRI sites, 50% will agree to participate in MRI. This results in a sample for the mechanistic study of 90 participants at baseline and, assuming 20% attrition, complete mechanistic data in 70 participants. This sample size has 80% power to detect a minimum between group effect size of 0.7SD on a standardised scale and 90% power for 0.8SD. In our previous observational study in TRS [43], we found a within-group effect after 12 weeks of clozapine of 0.7. Assuming there is no further change from prior treatment in the standard antipsychotic treatment arm on these measures over 12 weeks, we would have sufficient power to detect between-group effects of these magnitudes and expect the effect sizes to be greater in the younger population.

### Randomisation

Participants will be randomised 1:1 using an online system hosted by the King’s Clinical Trials Unit, using computer generated blocks of random sizes. The sample will be stratified by sex and age group (< 18 years, >  = 18 years).

### Blinding

The raters for primary outcome (PANSS) and CGI-S and CGI-I will be centralised and blinded to minimise observer bias. The participants, the treating team, and the rest of the trial team will not be blinded. There will be four blinded members of the study team: two research psychiatrists, one for adult participants and the other for child participants (each with appropriate training for that age group), the Chief Investigator (CI) and Co-Chief Investigator. They will not be informed which treatment arm the participants are in and will not enquire about the participant’s treatment or side effects.

### Data collection methods and management

A web based electronic data capture (EDC) system will be designed, using the InferMed Macro 4 system. The EDC will be created in collaboration with the trial analyst and the CI and maintained by the King’s Clinical Trials Unit for the duration of the project. It will be hosted on a dedicated server within King’s College London. The system is compliant with FDA 21 CFR part 11 and Good Clinical Practice (GCP). The CI will act as custodian for the trial data. Should a participant decide to withdraw from the study, all efforts will be made to report the reason for withdrawal as thoroughly as possible. Should a participant withdraw from study drug only, efforts will be made to continue to obtain follow-up data, with the permission of the participant.

### Ethics and regulatory approvals

The trial will be conducted in compliance with the principles of the Declaration of Helsinki (1996), the principles of GCP and in accordance with all applicable regulatory requirements including but not limited to the Research Governance Framework and the Medicines for Human Use (Clinical Trial) Regulations 2004, as amended in 2006 and any subsequent amendments. The trial has been approved by London—Dulwich Research Ethics Committee (REC reference: 22/LO/0723) and obtained Clinical Trial Authorisation (CTA) from the Medicines & Healthcare products Regulatory Agency (MHRA; CTA 22926/0007/001–0003).

### Statistical analysis

The Statistical Analysis Plan will be drafted within 6 months after recruitment starts and will be approved by the Data Monitoring Committee (DMC) and Trial Steering Committee (TSC) before the junior statistician sees any outcome data split by arm. The senior statistician will remain fully blind throughout the study.

We will report data in line with the CONSORT 2010 statement [57] showing attrition rates and loss to follow-up. The target estimand is the treatment policy estimand, and all primary and secondary analyses will be carried out following the intention to treat principle, incorporating data from all participants including those who do not complete treatment. Every effort will be made to follow up all participants in both arms for research assessments. The trial will be blind rated to minimize observer bias. The primary analysis will be conducted after the final 12-week follow-up assessment is completed for the final patient recruited into the study. The study team including CI and trial statisticians will be aware of the results of the primary analysis from this point. The remaining 24-and 52-week assessments will continue to be conducted blind to individual patient’s allocation. We will analyse the 24- and 52-week outcomes in a secondary analysis.

Analyses will be conducted in Stata version 17or later. Descriptive statistics within each randomized group will be presented for baseline values. For the primary analysis, the treatment effects on primary and secondary outcomes will be estimated using linear mixed models fitted to all outcome variables up to and including the 12-week assessment. Fixed effects will be sex, age (< 18, >  = 18) and duration of previous treatment (± 3 years), baseline assessment for the outcome under investigation, treatment, time, and time*treatment interactions. Participant will be included as a random intercept to account for repeated measures. Marginal treatment effects will be estimated for primary outcome (PANSS score at 12 weeks), and for PANSS scores at each other time point (6 weeks, 12 weeks), and reported separately as adjusted mean differences in scores between the groups with 95% confidence intervals and 2-sided p-values. For secondary outcomes the same approach will be followed using linear mixed models to estimate and report the treatment effect at each time point. Cohen’s D effect sizes will be calculated as the adjusted mean difference of the outcome divided by the sample standard deviation of the outcome at baseline. These will be displayed in a forest plot showing the treatment effects on the primary and the secondary outcomes at 12 weeks. For the secondary analysis including 24-and 52-week time points, we will repeat the linear mixed model approach with these additional outcomes in the response vector. We will report the estimated marginal treatment effects at 24-and 52-weeks.

Missing data on individual measures will be pro-rated if more than 80–90% (depending on questionnaire) of the items are completed; otherwise, the measure will be considered as missing. We will check for differential predictors of missing outcomes by comparing responders to non-responders on key baseline variables. Any significant predictors will be included in the analysis models in a sensitivity analysis. This accounts for missing outcome data under a missing at random assumption, conditional on the covariates included in the model. As a sensitivity analysis, we will assess whether treatment adherence is associated with missing data, and if it is associated, use inverse probability weights or multiple imputation to compare results.

A pre-specified subgroup analysis will test the treatment effect in children (age < 18 years) by estimating the effect in each group separately. If the final proportion of under 18 s is low, we may use a Bayesian subgroup analysis to increase precision of the effect in the groups. There are no planned interim analyses.

For the mechanistic study, descriptive statistics for biomarkers at baseline and 12 weeks will be presented by treatment group. We will test for between-group differences on peripheral cytokines, GSH, brain glutamate and rCBF at 12 weeks using appropriate generalised linear models. Mechanistic analyses will be based on causal mediation analysis and the primary outcome variable will be the PANSS total score at 12 weeks. We will estimate the direct and indirect effects of clozapine on total PANSS via the mechanistic measures by fitting models for the outcome with and without mediators using the difference in coefficients approach, extended for multiple mediators. We will adjust for baseline values of the mediators [58] baseline PANSS total score, and fixed effects of sex, age, duration and number of previous treatment trials [59].

### Economic evaluation

A detailed health economic analysis plan (HEAP) will be prepared by the trial health economists and approved by the Data Monitoring Committee (DMC) and Trial Steering Committee (TSC) before the trial health economist sees any data split by arm. The HEAP will be drafted before recruitment starts and will be approved before the junior health economist sees any outcome data split by arm. The senior health economist will remain fully blind until review of the first draft of the final economic analysis reports for checking when they will become fully unblinded and any future amendments to the HEAP will be made by them.

A within-trial cost-effectiveness analysis will be carried out, taking the NHS and social services perspective preferred by NICE, including relevant education-based health and social care services, given the age group. Service use will be collected in interview at baseline (covering the previous 3 months) and at the 6-, 12-, 24- and 52-week follow-up assessments (covering the period since last interview) using the Early Intervention Adult Service Use Schedule (EI-AD-SUS) [53]. The EI-AD-SUS was originally designed and successfully applied in populations of young people and young adults at risk of or with psychosis. Nationally applicable unit costs will be applied to all services (for example, NHS Reference Costs for hospital contacts, British National Formulary for medications, PSSRU Unit Costs of Health & Social Care for community-based services etc.) to estimate total costs per participant.

The primary economic evaluation will be a cost-utility analysis carried out at the 12-week follow-up, in line with the primary clinical analysis, with outcomes expressed in terms of quality adjusted life years (QALYs) calculated from the EQ-5D-Y [51], as preferred by NICE [60], and using the recommended area under the curve approach [61]. Secondary analyses will explore a) cost-effectiveness using the primary clinical measure of outcome (PANSS total score) at 12-weeks and b) cost-utility using QALYs at the 52-week follow-up. Given evidence to suggest the EQ-5D may not be particularly sensitive in psychosis populations [62], we will additionally include the Recovering Quality of Life-10items measure (ReQoL-10), a new generic self-reported outcome measure for use with people experiencing mental health difficulties, which may be more sensitive to change than the EQ-5D. The ReQoL is not appropriate as the main measure of effectiveness for the economic evaluation because it is not yet associated with preference weights to generate QALYs for use in cost-effectiveness analyses and it is currently considered suitable for people aged 16 and over. However, the inclusion of this brief measure will support exploration of the sensitivity of the EQ-5D in comparison to the ReQoL and the validity of the measure in young people under the age of 16.

Costs and QALYs will be presented as mean values by trial arm with standard deviations. Mean differences in costs and 95% confidence intervals will be obtained by non-parametric bootstrap regressions to account for the non-normal distribution commonly found in economic data [63]. Cost-effectiveness will be assessed using the net benefit approach and following standard approaches [64]. A joint distribution of incremental mean costs and effects for the two groups will be generated using bootstrapping to explore the probability that clozapine is the optimal choice compared to TAU, subject to a range of possible maximum values (ceiling ratio) that a decision-maker might be willing to pay for unit improvements in outcomes. Cost-effectiveness acceptability curves will be presented by plotting these probabilities for a range of possible values of the ceiling ratio [65]. These curves are the recommended decision-making approach to dealing with the uncertainty that exists around the estimates of expected costs and expected effects associated with the interventions under investigation and uncertainty regarding the maximum cost-effectiveness ratio that a decision-maker would consider acceptable. To provide more relevant treatment-effect estimates, all economic analyses will include adjustment for the variable(s) of interest and baseline covariates [66], which will be prespecified and in line with the clinical analyses. Complete case analyses will be carried out with the impact of missing data explored in sensitivity analyses. The pattern of missing data and the plausibility of assuming the data are Missing at Random will be examined for missing cost and EQ-5D-Y tariff values. Any variables predictive of missingness or predictive of response (at *P* < 0.1) will be included in the equation to impute missing values.

### Safety

Participants will be asked at each visit from consent onwards to report any suspected adverse reactions. Any suspected adverse events will be recorded from consent visit to end of trial. Any suspected adverse events recorded will be explored again at each visit thereafter. Events or reactions listed in the Summary of Product Characteristics (SmPC) do not need to be reported unless they fulfil seriousness criteria.

All Serious adverse Event (SAE), Serious Adverse Reaction (SAR) or Suspected Unexpected Serious Adverse Reaction (SUSAR), excepting those specified in the protocol as not requiring reporting, will be reported immediately by the CI (and certainly no later than 24 h) to the KHP-CTO in accordance with the current Pharmacovigilance Policy. The KHP-CTO will report SUSARs to the regulatory authority (MHRA).

### Data monitoring and auditing

A DMC will be established to review accruing data and safety information, reporting to the TSC. Independent membership will include an adult psychiatrist, a child and adolescent psychiatrist, and a clinical trial statistician. The Investigator will permit trial-related monitoring, audits, REC review, and regulatory inspections by providing the Sponsor, Regulators and REC direct access to source data and other documents.

### Availability of data and materials

The datasets used and/or analysed during the current study will be available from the corresponding author on reasonable request.

## Discussion

The CLEAR trial raises ethical, practical and organizational challenges, and we hope our design solutions will help improving inclusivity in research in severe mental illness.

We are aware recruitment for this trial will be challenging, as prevalence of TRS in young people is low, and this population might be hard to enrol in research. In fact, people with severe mental illness, especially TRS, are often not included in trials due to the impact of their symptoms on capacity, and there is evidence of systemic exclusion from research leading to lack of strong generalisable results in TRS research [67, 68]. This is even more true in young people, especially <16, whose consent to participate needs additional consideration. In the CLEAR trial, there are three ethical aspects that require further caution regarding consent:

- Some of the participants may lack capacity to give informed consent to the trial
- Some of the participants may be detained in hospital under the Mental Health Act, which will usually entail a requirement to stay in hospital and may also include a requirement to take treatment. It should be noted that many patients detained under the Mental Health Act retain the capacity to consent to research [69]
- Some participants will be under 16 and thus prohibited under the Medicines for Human Use (Clinical Trials) Regulations from giving consent to participate in a clinical trial.

All three of these issues can be addressed by the appointment of a legal representative. Under the UK Clinical Trials Regulation No 536/2014, a relative or friend may act as legal representative, who must decide whether the person lacking capacity should participate in the trial based on what they would have wanted had they the capacity to choose for themselves, their ‘presumed will’. The legal representative will be given the opportunity to understand the objectives, risks, and inconveniences. We hope that this solution will maximise inclusion in the trial and will raise awareness on the sensitive matter of inclusion of severely unwell people with schizophrenia in research.

The CLEAR trial is designed to be as close to real-world settings as possible to optimise the acceptability by participants and the generalisability of the results. In the trial, only the raters will be blinded, neither clinicians nor participants, because of the need for individualised treatment dose and management of drug-specific side effects. Furthermore, as clozapine treatment needs strict weekly blood monitoring, we believe it would be unethical to have this requirement in TAU without any clear clinical indication.

All the trial participants will be able to change treatment after 12 weeks, and those in the TAU arm could at this time start clozapine if deemed appropriate by the treating team. We will continue assessing all participants in the follow-up period, when we will record their current treatment as well as service use. We believe this design is the most ethical solution as it will not prevent TRS patients from the recommended treatment in the longer term.

## Conclusion

Overall, we believe that the CLEAR trial will uniquely contribute to knowledge about the efficacy, safety, and mechanism of action of clozapine in young people with TRS, providing strong evidence to reinforce clinical guidance.

## Data Availability

Not applicable.

## Abbreviations

AE: Adverse event
AR: Adverse reaction
CGI: Clinical Global Impression scale
CRS: Clinician Rating Scale
DAI-10: Drug Attitude Inventory
DMC: Data Monitoring Committee
EI-AD-SUS: Early Intervention Adult Service Use Schedule
EQ-5D-Y: Youth version of the EQ-5D-3L (5 dimensions, 3 levels) measure of health-related quality of Life
GASS-C: Glasgow Antipsychotic Side-effect Scale for Clozapine
GCP: Good Clinical Practice
PANSS: Positive and Negative Syndrome Scale
ReQoL-10: Recovering Quality of Life for users of mental health services-10 items
SAE: Serious adverse event
SAR: Serious adverse reaction
SUSAR: Suspected unexpected serious adverse reaction
TRS: Treatment resistant schizophrenia
TSC: Trial Steering Committee
